# Supplementary material for: Uncertainty Shapes Neural Dynamics in Motor Cortex During Reaching
Source: bioRxiv. 2025 Oct 15:2025.10.14.682390. Preprint. [Version 1] doi: 10.1101/2025.10.14.682390 (PMC12632930; doi:10.1101/2025.10.14.682390)
Supplement: 1 [file NIHPP2025.10.14.682390v1-supplement-1.pdf]

bioRxiv preprint doi: https://doi.org/10.1101/2025.10.14.682390; this version posted October 15, 2025. The copyright holder for this preprint (which was not certified by peer review) is the author/funder, who has granted bioRxiv a license to display the preprint in perpetuity. It is made available under aCC-BY 4.0 International license.

SUPPLEMENTAL INFORMATION

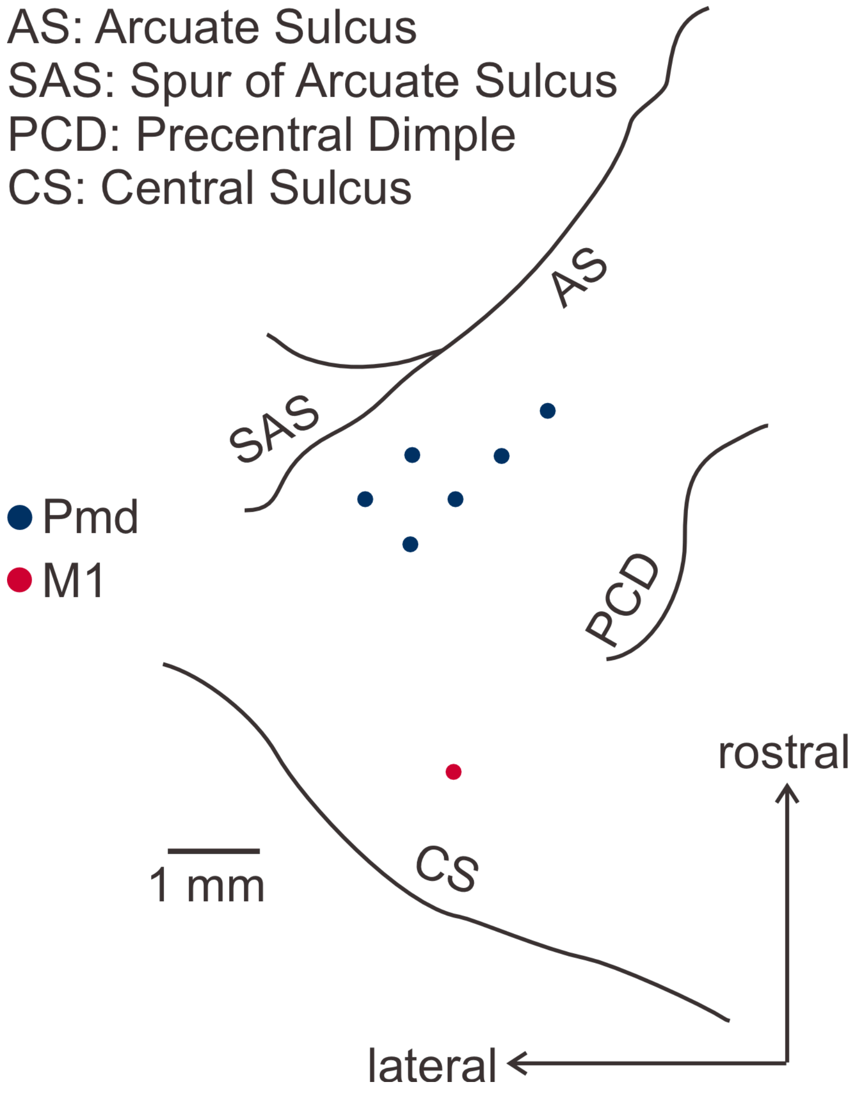

<u>Supplementary Figure 1</u>. Recording sites across eight sessions. The M1 recording site was recorded from twice.

bioRxiv preprint doi: https://doi.org/10.1101/2025.10.14.682390; this version posted October 15, 2025. The copyright holder for this preprint (which was not certified by peer review) is the author/funder, who has granted bioRxiv a license to display the preprint in perpetuity. It is made available under aCC-BY 4.0 International license.

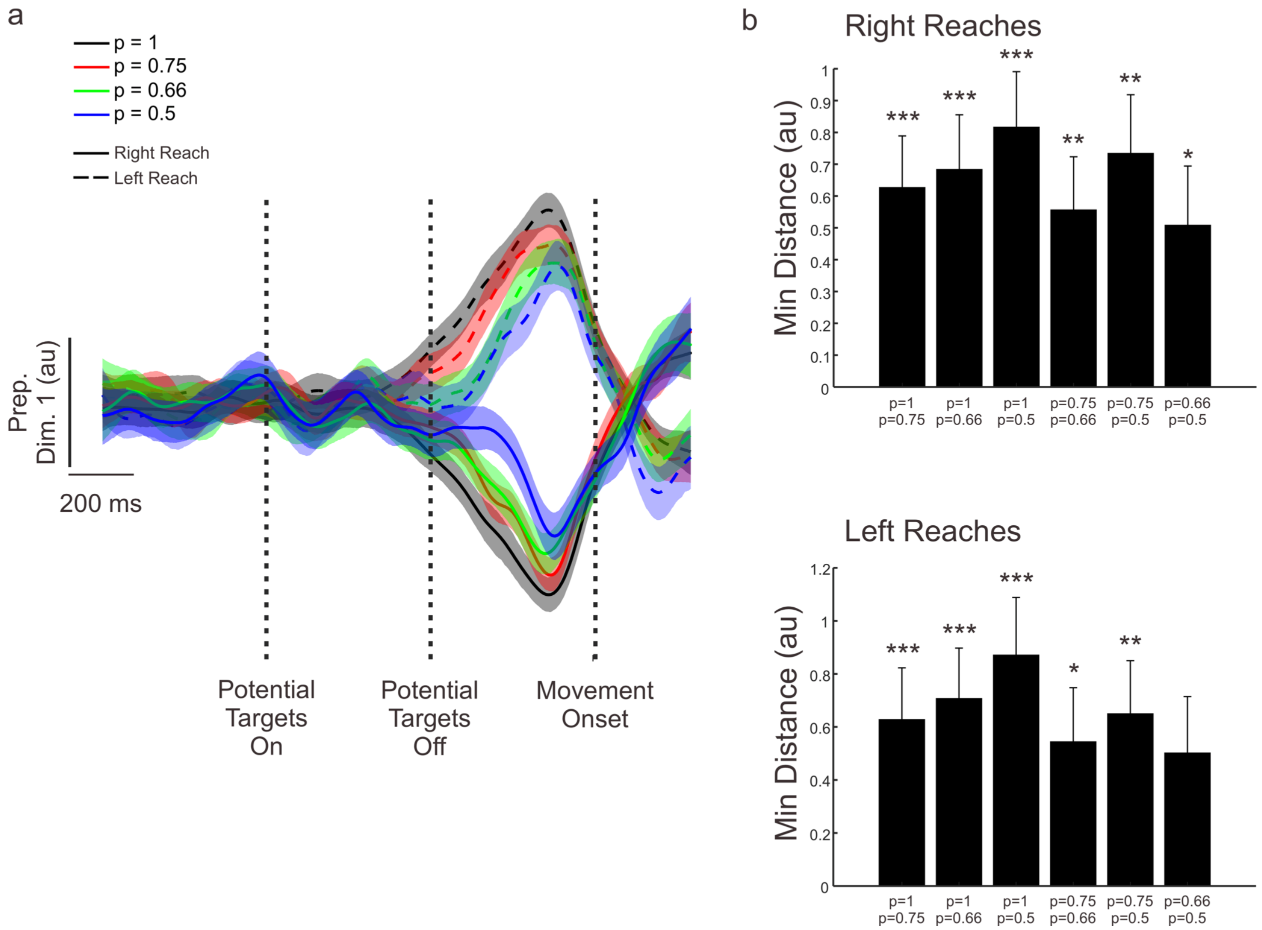

Supplementary Figure 2. Statistical comparison of preparatory dynamics. a) Mean and 95% confidence interval of preparatory dynamics in PMd along the first dimension. b) Median minimum distance between pairs of preparatory states at peak occupancy estimated using bootstrapping. The error bars represent the 5th and 95th percentile of the bootstrapped minimum distances. The stars represent the bootstrap significance levels (- p < 0.05, ** - p < 0.01, *** - p < 0.001).*
